# Dietitians as agents of change to increase legume consumption: a randomized controlled trial of a behavioral intervention

**DOI:** 10.3389/fnut.2025.1713719

**Published:** 2026-01-28

**Authors:** Orit Ofir, Aliza H. Stark, Wiessam Abu Ahmad, Yael Bar-Zeev

**Affiliations:** 1 The Robert H. Smith Faculty of Agriculture, Food and Environment, School of Nutritional Sciences, Institute of Biochemistry, Food Science and Nutrition, The Hebrew University of Jerusalem, Rehovot, Israel; 2 Braun School of Public Health and Community Medicine, Faculty of Medicine, Hebrew University of Jerusalem-Hadassah Medical Centre, Jerusalem, Israel

**Keywords:** agents of change, dietetic practice, legume consumption, online learning, sustainable diets

## Abstract

**Introduction:**

Health and environmental benefits of daily legume consumption are reflected in Israeli Dietary Guidelines. However, legume intake fails to meet recommendations. Dietitians may be effective agents of change for promoting legume consumption. This study evaluates an evidence and theory-based, multi-component, intervention aimed to improve Israeli dietitians’ legume counseling practices, knowledge, attitudes and personal intake.

**Methods:**

A randomized controlled trial (May–September 2023) was conducted among dietitians who actively counsel patients. The intervention included a prerecorded webinar followed by small-group workshops and provision of brochures for patients, alongside a professional guide on legume counseling for dietitians. Data regarding legume knowledge, attitudes, counseling practices and personal intake were collected at baseline and 3 months post-intervention. Controls were wait-listed to receive the intervention. The primary outcome was self-reported proportion of patients recommended to consume legumes daily (1–5 Likert scale: (1) none; (2) ≤25%; (3) 26–50%; (4) 51–75%; and (5) 76–100%). A repeated measures mixed-design model, chi-square tests and pairwise odds ratio tests were utilized for the analysis.

**Results:**

Overall, 213 dietitians participated (Intervention: *n* = 109, Control: *n* = 104). The proportion of dietitians in the intervention group recommending to 76–100% of their patients to consume legumes daily increased from 32% (baseline) to 51% (follow-up); compared to 25 and 27%, respectively, in the controls. In the repeated measures model, recommending daily legume consumption improved significantly in both the intervention group (3.73 ± 1.1 to 4.28 ± 0.86, *p* = 0.001) and the control (3.67 ± 0.98 to 3.88 ± 0.92, *p* = 0.03), with a higher increase in the intervention group (*p* = 0.014). Knowledge and attitudes improved significantly in the intervention group (*p* < 0.001) but not for controls, except in the attitude score regarding sustainability (*p* = 0.026). Personal legume consumption improved significantly only in the intervention group, who had higher odds of increasing legume intake to at least twice a week [OR 2.81 (95%CI: 1.10–8.11)].

**Discussion:**

An online intervention significantly improved dietitians’ knowledge, attitudes, counseling practices regarding legume consumption and personal intake. Utilizing dietitians’ counseling might be a viable approach for promoting consumption of sustainable diets.

## Introduction

There is universal consensus regarding legumes’ key role in sustainable and nutritious diets (1–5). Daily consumption of legumes (beans, lentils, peas and soy; 75 g/day, equivalent to ~2/3 cup) is a central component in the EAT-Lancet Commission Planetary Health Diet (5). Legumes are a good source of protein and iron (6). In comparison to animal protein sources, they are low in saturated fat while high in dietary fiber and phytochemicals (6). Health benefits associated with legumes include prevention of cardiovascular diseases (7–10), diabetes (11, 12), colorectal cancer (10), and a decrease in all-cause mortality (13). Legume intake is acknowledged as a vital factor in global endeavors to deal with the environmental crisis by decreasing greenhouse gas emissions and the reduced use of water, land and fuel compared to animal protein production (5, 14, 15). The 2025 Advisory Committee for the Dietary Guidelines for Americans 2025–2030 (16), and national guidelines from various countries such as Canada (17) and the United Kingdom (18) recommend legumes as a preferred protein source. However, actual consumption levels worldwide are low (19, 20). Current Israeli dietary guidelines recommend daily legume consumption (21). Data from the 2014–2016 national nutrition survey, showed that legumes were consumed by 31% of Israelis with a mean intake of ~0.25 cup/day (Israel Ministry of Health, 2022, secondary data analysis, unpublished data). Thus, there is a distinct need to promote legume incorporation into the daily diet. To the best of our knowledge, interventions aimed at increasing legume consumption have been conducted only among the general public, predominately in low-income countries (22–26). Three recent interventions were performed in high-income countries; however, the sample size was small and none were randomized control trials (27–29). In comparison to interventions among the general public, training health professionals as “agents of change” could potentially reach a larger population, and may be more easily replicated across countries and cultures (30). Online training might also further increase reach (31). Thus, dietitians could be an ideal target population due to their prominent role in affecting consumption patterns (32–34). A previous survey among Israeli dietitians found their legume counseling and consumption practices were well below current guidelines (35). Less than a third of Israeli dietitians recommended to most or all of their patients to consume legumes daily, and only about 5% reported following the recommendation themselves. Factors that were associated with recommending legumes to patients were higher personal legume consumption, more favorable perceptions regarding barriers toward legume consumption, more favorable attitudes toward the importance of legume counseling and working in an outpatient setting (35). Based on these results (35), an online, multi-component, theory and evidence-based, behavioral intervention was developed. This study aimed to evaluate the impact of the intervention on Israeli dietitians’ knowledge, attitudes and counseling practices regarding legumes, including improving their personal consumption patterns.

## Methods

### Study design

**Figure 1:**
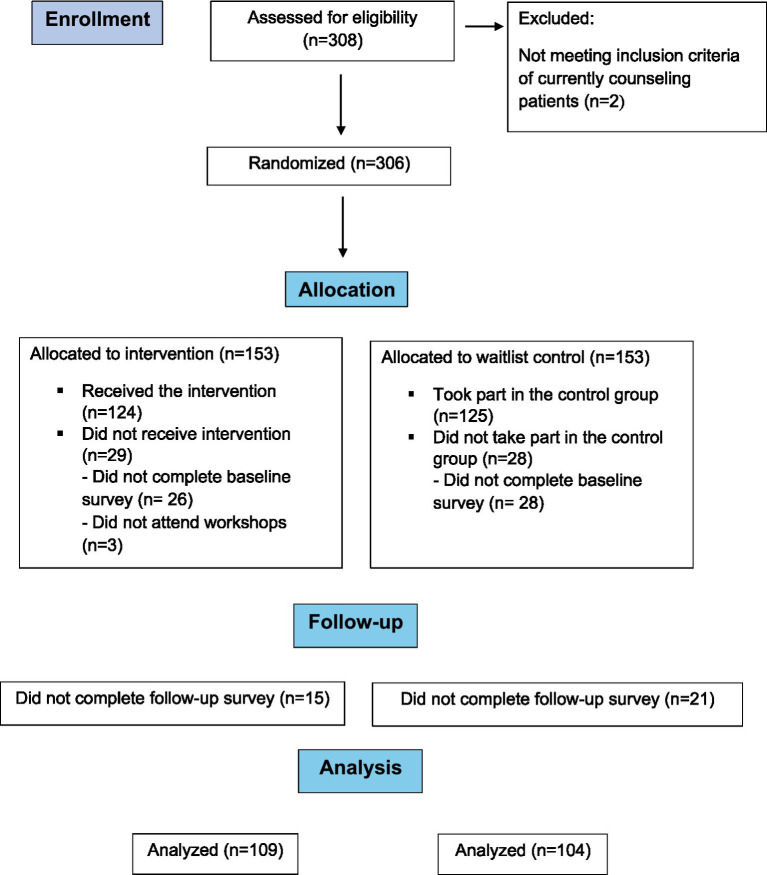
Flow chart of study participants.

A randomized controlled trial was carried out (May–September 2023), with participants randomly assigned, by the first author (OO) to either the control or intervention group using simple randomization based on the parity of their enrollment number (i.e., even vs. odd). All participants completed a baseline survey (May 2023), after which the intervention group received the intervention (May–June 2023), while the control group was wait-listed to participate at a later date. Both groups were followed for 3 months at which time they completed a follow-up survey (September 2023) (Figure 1). After completing the follow-up survey, the control group had an opportunity to access the intervention. The study was approved by the Hebrew University of Jerusalem-Robert H. Smith, Faculty of Agriculture, Food and Environment Ethics Committee (AGHS/May-2.23).

### Participants

Eligible participants were Israeli dietitians who actively counsel patients. Dietitians that work solely in the public health sphere or in the food industry were excluded.

An invitation to participate in the study was dispersed via: (1) emailing dietitians who completed a preceding survey (35) and agreed to be contacted in the future (*n* = 186); (2) electronic mailing list of the Israeli Dietetic Association (ATID); (3) WhatsApp groups of the nutrition division of the Israeli Ministry of Health; (4) personal communications with head dietitians in the four Israeli Health Maintenance Organizations (HMOs); and (5) relevant Facebook groups for dietitians. In order to aid recruitment and minimize dropout, a drawing for three ATID professional courses, three annual ATID conference registration fees and two electronic tablets, was carried out among participants who completed the follow-up survey (35).

Informed consent was obtained upon enrollment. After randomization, all participants received an email containing a link to the baseline survey.

### Intervention

The intervention design was guided by the Theoretical Domains Framework (TDF) and the COM-B Behavior Change Model (36–38). Both the TDF and the COM-B are validated and integrative models of behavior change and have been applied across a wide scope of clinical situations (39).

The intervention was carried out by an experienced dietitian with proficiency in plant-based diets and specifically counseling regarding legume consumption (the first author), and included:

- A prerecorded 45-min webinar presenting nutritional, health, food security and environmental benefits of legumes.
- Small-group Zoom workshops to enhance skills for overcoming common barriers to legume consumption. Nine workshops were carried out, with an average of 13 participants, lasting about 90 min; with attendance monitored. In order to ensure quality and consistency across all nine workshops, a single individual (first author) carried out all the sessions. The same presentation was used throughout, and slides were sent to the participants prior to each workshop. Additionally, following delivery of the workshops, the lecture given in the workshops was adjusted to incorporate additional topics that repeatedly arose. A recording of this comprehensive lecture was sent to the participants.
- A professional guide on legume counseling for dietitians, and brochures to be distributed to patients (40). The brochure content is featured in Supplementary File 1a. Each participant received by mail one hard copy of the professional guide and 80 patient brochures, and via email PDF versions of the guide and the brochures.
- An email reminder (August 2023) was sent to the intervention group recommending watching the webinar and workshop recording for a second time. The rationale was to allow participants to further assimilate the information presented and provide additional highlights on legume dishes that are more compatible in the summer (e.g., salads, omelets).

### Measures and outcomes

The baseline survey (Supplementary File 1b) was based on a previous survey (35). Questions were adapted from previous surveys focusing on legumes among dietitians (41, 42) and the general public (43, 44). Questions regarding attitudes toward counseling were based on the TDF (37, 38) and the COM-B model (36).

The survey included six sections:

(a) Socio demographic and professional characteristics: including age, sex, education, primary workplace, years in practice, average number of patients per week, average number of meetings per patient, whether providing virtual counseling and area of expertise (e.g., obesity); Personal diet pattern was categorized as omnivore, flexitarian, vegetarian + vegan. Awareness of the current legume guidelines was assessed by inquiring whether the participant was aware of the recommendation of daily legume intake. (b) Legume counseling to patients: The primary outcome was measured on a 1–5 Likert scale as the proportion of patients that the dietitian recommends to consume legumes *daily*, dichotomized to 76–100 and 75% or less. Two additional questions used the same Likert scale, and asked about: (1) the proportion of patients that the dietitian recommended to *increase* legume consumption in general, and (2) legume counseling practices specifically for vegans\ vegetarians\ flexitarians. (c) Knowledge was measured using seven true/false/do not know statements pertaining to health, nutritional and ecological attributes of legumes (e.g., “Legumes may help reduce LDL cholesterol levels”). A composite knowledge score was constructed by summing the number of correct answers (scale 0–7). ‘Do not know’ answers were considered as incorrect. (d) Attitudes toward legume counseling were measured with ten statements, using a 5-point Likert scale of “strongly disagree” (1) to “strongly agree” (5). A principal factor analysis (varimax rotation) was performed to reduce the statements to three factors: “Resources, confidence and knowledge” (“Importance, effectiveness and time” and “Sustainability”) (Supplementary File 1b). For each factor, a mean composite attitude score was created. Negatively framed statements were reversed such that all scores would be in the same direction, i.e., a higher mean composite score corresponds to a higher positive attitude. Cronbach’s *α* was 0.673, 0.544, 0.614, respectively. (e) Legume perceptions were assessed by ten statements on legume nutritional and health benefits and barriers for consumption using the same Likert scale as above. A similar process was implemented, reducing the ten statements to three factors: “Adequate protein and iron source” “Management of consumption barriers” (and “Weight and glucose management”) (Supplementary File 1b). For each factor, a mean composite attitude score was created. Cronbach’s α for the three factors was 0.680, 0.668, 0.440, respectively. (f) Personal legume consumption was measured with the question: “How often do you consume legumes (not including soy milk in coffee)?” dichotomized to twice a week or more, and once a week or less.

The follow-up survey included the same questions, without the sociodemographic and professional characteristics section. The intervention group survey included an additional section evaluating the training program. The participants were asked to rate [5-point Likert scale, “to a small extent” (1) to “to a great extent” (5)] the entire program and each of its components (i.e., webinar, workshop, dietitian guide, printed brochure for patients, digital brochure and recipe links) across three parameters: contribution to improving legume counseling effectiveness (i.e., leading to an increase in legume intake among patients), compatibility to the population the dietitian treats, and general satisfaction. Participants were also asked about their use of the program components (e.g., webinar completion, brochure and professional guide use).

### Sample size

A sample size of 248 participants was determined to be sufficient to detect an increase of 20% in the proportion of dietitians in the intervention group that recommend 76–100% of their clients to consume legumes daily (assuming a baseline counseling rate of ~30%, based on our previous cross-sectional survey) (35) with 80% power and a 0.05 significance level, factoring in a 20% dropout rate.

### Statistical analysis

Participants were included in the analyses if they had completed both baseline and follow-up surveys. A descriptive analysis was performed; categorical variables were reported as frequencies and percentages, and continuous variables as means and standard deviations. Bivariate analysis was conducted using the Pearson’s Chi-squared test or the Fisher’s exact test as appropriate for categorical variables, and Welch Two Sample t-test for continuous variables. Legume counseling and personal consumption measures were dichotomized into two categories: 76–100% vs. 75% or less, and “twice a week or more” vs. “once a week or less,” respectively. The data were then organized into contingency tables. Pairwise comparisons were conducted on 2 × 2 tables derived from the overall contingency table using the odds ratio test. A repeated measures model was performed to test differences in legume counseling measures as continuous variables, and in knowledge, perceptions and attitudes toward legume counseling, within and between the study groups over time and group by time interaction. Benjamini-Hochberg correction was applied to control for multiple comparisons. The level of significance for all tests was set to 0.05. All statistical analyses were performed using whole case analysis with RStudio, version 2023.01.1.

## Results

### Participant characteristics

A total of 308 dietitians enrolled in the study, of which 252 filled out the baseline survey and were randomized to the intervention group (*n* = 127) or waitlist control group (*n* = 125) (Figure 1). Three participants in the intervention arm did not attend the online workshops, thus not completing the program. The follow-up survey was answered by *n* = 213 (84.5%) participants which were included in the final analysis [intervention group, *n* = 109 (85.8%); control group, *n* = 104 (83.2%)]. The final sample was comprised of 98% (*n* = 208) women, with a mean age of 41.2 ± 9.1 years. Mean years in practice were 13 ± 9.4 with 46% (*n* = 98) of respondents holding graduate degrees. Main workplaces were HMOs (40%, *n* = 85), hospitals (27%, *n* = 58) and private clinics (19%, *n* = 41) (Table 1). The most common areas of expertise were obesity (69%, *n* = 147), diabetes (51%, *n* = 109) and healthy eating promotion (36%, *n* = 77). Only 42% (*n* = 89) of the dietitians were aware of the national dietary guidelines’ recommendation of daily legume intake. No significant differences were found between the study groups in any of their sociodemographic and professional characteristics (Table 1).

**Table 1:** Participants’ socio-demographic and work-related characteristics (*n* = 213).

Variable	Total sample *n* = 213 (*n*, %)	Intervention group *n* = 109 (*n*, %)	Control group *n* = 104 (*n*, %)	*p*-value*
Sex (% women)	208 (98%)	107 (98%)	101 (97%)	0.677
Age (mean ± SD)	41.2 ± 9.1	40.3 ± 8.7	42.1 ± 9.5	0.16
Education – Master degree or higher	98 (46%)	46 (42%)	52 (50%)	0.254
Main workplace				
Hospital	58 (27%)	27 (25%)	31 (30%)	0.821
Health Maintenance Organization	85 (40%)	44 (41%)	41 (39%)	
Private clinic	41 (19%)	23 (21%)	18 (17%)	
Other^1^	28 (13%)	14 (13%)	14 (13%)	
Years in practice (mean ± SD)	13 ± 9.4	12.5 ± 9.4	13.6 ± 9.5	0.417
Number of patients per week (mean ± SD)	28.6 ± 17.8	30 ± 18.7	27.2 ± 16.8	0.266
Number of meetings per patient (mean ± SD)	8.5 ± 8.2	8.8 ± 8.7	8.3 ± 7.7	0.678
Does not provide virtual counseling	49 (23%)	23 (21%)	26 (25%)	0.936
Awareness of current legume guidelines	89 (42%)	41 (38%)	48 (47%)	0.185
Personal diet pattern				
Omnivore	88 (41%)	43 (39%)	45 (43%)	0.756
Flexitarian	82 (38%)	42 (39%)	40 (38%)	
Vegetarian+ vegan	43 (20%)	24 (22%)	19 (18%)	

*The *p*-values presented in the table are the original values before adjustment for multiple comparisons. The adjusted *p*-values are between 0.903–0.964. ^1^Other: retirement home (*n* = 9, 4.2%), public health (*n* = 8, 3.8%), medical clinic (*n* = 6, 2.8%), university/research institute (*n* = 4, 1.9%) and gym (*n* = 1, 0.5%). Missing: main workplace *n* = 1, number of patients per week *n* = 4, number of meetings per patient *n* = 20, awareness of the current legume guidelines *n* = 1.

### Baseline counseling, knowledge, perceptions, attitudes and personal consumption

Less than a third (29%, *n* = 61) of the dietitians reported recommending to 76–100% of their patients to consume legumes daily, and nearly half (47%, *n* = 100) recommended to increase legume consumption in general (Table 2). The mean knowledge score for the total sample was 5.17 ± 1.21. There was a high level of agreement for the total sample for the attitudes regrading ‘sustainability’ and ‘importance, effectiveness and time’ (4.17 ± 0.78, 4.17 ± 0.55, respectively) and for the perceptions regarding ‘adequate protein and iron source’ and ‘weight and glucose management’ (4.41 ± 0.57, 4.35 ± 0.52, respectively). A lower level of agreement was found for the ‘resources, confidence and knowledge’ attitude (3.36 ± 0.73) and a low level of agreement was found for the ‘management of consumption barriers’ perception (2.77 ± 0.72). No significant differences were found between the study groups in any of these baseline variables (Table 3, *p* groups >0.1 for all). Almost a quarter [22% (*n* = 46)] of the sample reported consuming legumes at least four times a week, but only 5.2% (*n* = 11) reported consuming legumes daily, with no significant differences between the study groups (Table 2).

**Table 2:** Changes in legume recommendations and personal consumption in the intervention and control groups over time, (*n* = 213).

Variable	Baseline				Follow up			
	Total sample *n* = 213 (*n*, %)	Intervention group *n* = 109 (*n*, %)	Control group *n* = 104 (*n*, %)	*p*-value*	Total sample *n* = 213 (*n*, %)	Intervention group *n* = 109 (*n*, %)	Control group *n* = 104 (*n*, %)	*p*-value*
Recommending their patients to consume legumes daily								
To 76–100% of patients	61 (29%)	35 (32%)	26 (25%)	0.903	84 (39%)	56 (51%)	28 (27%)	0.001
To 75% or less of patients	151 (71%)	73 (68%)	78 (75%)		129 (61%)	53 (49%)	76 (73%)	
Recommending their patients to increase legume intake								
To 76–100% of patients	100 (47%)	53 (49%)	47 (45%)	0.903	119 (56%)	71 (66%)	48 (46%)	0.021
To 75% or less of patients	113 (53%)	56 (51%)	57 (55%)		93 (44%)	37 (34%)	56 (54%)	
Recommending their vegans, vegetarians and flexitarians patients to consume legumes daily								
To 76–100% of patients	174 (82%)	88 (81%)	86 (83%)	0.989	184 (87%)	87 (84%)	97 (89%)	0.370
To 75% or less of patients	39 (18%)	21 (19%)	18 (17%)		29 (13%)	22 (16%)	7 (11%)	
Personal legume consumption								
At least 4 times a week	46 (22%)	28 (26%)	18 (17%)	0.154	68 (32%)	45 (41%)	23 (22%)	0.012
2–3 times a week	79 (37%)	40 (37%)	39 (38%)		68 (32%)	36 (33%)	32 (31%)	
Once a week	47 (22%)	18 (17%)	29 (28%)		40 (19%)	15 (14%)	25 (24%)	
3 times a month or less	41 (19%)	23 (21%)	18 (17%)		37 (17%)	13 (12%)	24 (23%)	

*The *p*-values presented in the table are after adjustment for multiple comparisons.

**Table 3:** Changes in legume counseling, knowledge, attitudes toward legume counseling, and legume perceptions, in the intervention and control groups over time, *n* = 213.

Outcome variable	Group	Baseline	Follow-up	*p* time^a^	*p* time*group^b^
Recommending daily legume intake	Intervention	3.73 ± 1.10	4.28 ± 0.86	<0.001	0.014
	Control	3.67 ± 0.98	3.88 ± 0.92	0.03	
	*p* groups^c^	0.684	0.002		
Recommending to increase legume intake	Intervention	4.14 ± 1.05	4.58 ± 0.64	<0.001	0.015
	Control	4.16 ± 0.94	4.32 ± 0.71	0.07	
	*p* groups^c^	0.858	0.005		
Knowledge	Intervention	5.39 ± 1.10	6.35 ± 0.75	<0.001	<0.001
	Control	5.27 ± 1.32	5.29 ± 1.24	0.855	
	*p* groups^c^	0.486	<0.001		
Attitudes toward legume counseling					
Resources, confidence and knowledge	Intervention	3.33 ± 0.74	4.6 ± 0.48	<0.001	<0.001
	Control	3.41 ± 0.72	3.36 ± 0.68	0.427	
	*p* groups^c^	0.445	<0.001		
Importance, effectiveness and time	Intervention	4.15 ± 0.56	4.35 ± 0.53	<0.001	
	Control	4.19 ± 0.54	4.19 ± 0.48	0.914	
	*p* groups^c^	0.591	0.024		
Sustainability	Intervention	4.24 ± 0.73	4.45 ± 0.67	<0.001	
	Control	4.1 ± 0.82	4.24 ± 0.69	0.026	
	*p* groups^c^	0.214	0.026		
Legume perceptions					
Adequate protein and iron source	Intervention	4.44 ± 0.57	4.73 ± 0.40	<0.001	0.322
	Control	4.38 ± 0.57	4.44 ± 0.50	0.161	
	*p* groups^c^	0.456	<0.001		
Management of consumption barriers	Intervention	2.78 ± 0.71	3.16 ± 0.58	<0.001	
	Control	2.77 ± 0.74	2.84 ± 0.71	0.245	
	*p* groups^c^	0.866	<0.001		
Weight and glucose management	Intervention	4.33 ± 0.53	4.54 ± 0.51	<0.001	
	Control	4.37 ± 0.51	4.42 ± 0.49	0.17	
	*p* groups^c^	0.593	0.087		

^a^*p*-value for changes over time, from baseline to follow up, in each group; ^b^*p*-value for interaction between the trend of change over time and the group effect; ^c^*p*-value for between-groups differences (intervention vs control group) in each time point. Legume counseling score were structured as a 1–5 Likert scale of “none” (1) to “76–100%” (5). The knowledge score was constructed by summing the number of correct answers (scale of 0–7); the attitudes and perceptions scores were structured as a 5-point Likert scale of “strongly disagree” (1) to “strongly agree” (5). *The *p*-values presented in the table are adjusted for multiple comparisons. **The composite score for the whole factor uses a converted score for these negative statements.

### Intervention effectiveness

#### Legume intake recommendations to patients

For the first outcome ‘recommending daily legume consumption’, the proportion of dietitians in the intervention group recommending this to 76–100% of their patients, increased from 32% (*n* = 35) at baseline to 51% (*n* = 56) post intervention; compared to 25% (*n* = 26) and 27% (*n* = 28), respectively, in the control group (Table 2). In the pairwise comparisons (Supplementary File 2 in Table S1), 23.1% (*n* = 25) of the intervention group increased recommending daily legume consumption to 76% or more of patients compared to only 16.3% (*n* = 17) in the controls. However, the odds of increasing counseling to 76% or more of patients were not significantly different between the groups [OR 1.54 (95% CI: 0.78–3.1)]. A lower proportion of the intervention group decreased their counseling to less than 76% of their patients [4.6% (*n* = 5) intervention vs. 14.4% (*n* = 15) control; OR 0.29 (95% CI 0.09–0.77)]. A higher proportion of the intervention group maintained counseling for 76% or more of patients compared to the controls [27.8% (*n* = 30) intervention vs. 10.6% (*n* = 11) control; OR 3.25 (95% CI: 1.57–7.18)] (Supplementary File 2 in Table S1). In the repeated measures model ‘recommending daily legume consumption’ improved significantly in both the intervention group (3.73 ± 1.1 to 4.28 ± 0.86, *p* = 0.001) and the controls (3.67 ± 0.98 to 3.88 ± 0.92, *p* = 0.03), with a significant interaction between time and group, i.e., the improvement in the intervention group was significantly higher in comparison to the controls (Table 3, *p* time*group = 0.014). For the second outcome ‘recommending to increase legume intake’, the proportion of dietitians in the intervention group recommending this to 76–100% of their patients, increased from 49% (*n* = 53) at baseline to 66% (*n* = 71) post intervention; compared to 45% (*n* = 47) and 46% (*n* = 48), respectively, in the controls (Table 2). In the pairwise comparisons, a similar pattern to the first outcome was found (Supplementary File 2 in Table S1). In the repeated measures model, ‘recommending to increase legume intake’ improved significantly in the intervention group (4.14 ± 1.05 to 4.58 ± 0.64, *p* ≤ 0.001) but not in the controls (4.16 ± 0.94 to 4.32 ± 0.71, *p* = 0.07).

#### Knowledge, legume perceptions, and attitudes toward legume counseling

The scores of knowledge, attitudes toward legume counseling and legume perceptions increased significantly at follow-up in the intervention group (*p* < 0.001 for all) (Table 3). The ‘Resources, confidence and knowledge’ attitude improved the most and increased by 1.3 points. The included statement “I have adequate didactic resources to counsel patients regarding legume consumption” received the lowest level of agreement (2.3 ± 1.1) out of all attitudes and perceptions at baseline and improved the most at follow-up (4.6 ± 0.6) (data not shown). No significant differences were found between the baseline and follow-up scores in the control group, apart from an increase in the attitude score regarding sustainability (4.1 ± 0.82 vs. 4.24 ± 0.69, *p* = 0.026). The intervention groups’ follow-up scores were significantly higher than those of the controls for all variables (Table 3, *p* groups^c^ < 0.05 for all), with the exception of a non-significant trend toward higher scores in perceptions regarding weight and glucose management (4.54 ± 0.51 intervention vs. 4.42 ± 0.49 control, *p* = 0.087). The interaction between the trend of change over time and the group effect was significant for knowledge and attitudes toward legume counseling (Table 3, *p* time*group^b^ < 0.001 for both) but not for legume perceptions (*p* = 0.322).

#### Personal legume consumption

The proportion of dietitians in the intervention group consuming legumes at least twice a week, increased from 62.3% (*n* = 68) at baseline to 75% (*n* = 81) post intervention; compared to a decrease from 54.9% (*n* = 57) to 52.6% (*n* = 55) in the control group (Table 2). In the pairwise comparisons, the intervention group had lower odds of maintaining a low legume intake of once a week or less [OR 0.46 (95% CI: 0.25–0.82), *p* = 0.04] and there was a trend toward higher odds of increasing legume intake to at least twice a week [OR 2.81 (95% CI: 1.1–8.11), *p* = 0.078] (Table 4).

**Table 4:** Pairwise comparisons of dietitians’ personal legume consumption, *n* = 213.

Personal legume consumption	Intervention group *n* = 109 (*n*, %)	Control group *n* = 104 (*n*, %)	OR (95%)	*p*-value*
Remaining in at least twice a week	65 (59.6%)	49 (47.1%)	1.66 (0.97–2.86)	0.091
Improving to at least twice a week	16 (14.7%)	6 (5.8%)	2.81 (1.10–8.11)	0.078
Declining to once a week or less	3 (2.8%)	8 (7.7%)	0.34 (0.07–1.21)	0.118
Remaining in once a week or less	25 (22.9%)	41 (39.4%)	0.46 (0.25–0.82)	0.04

*The *p*-values are adjusted for multiple comparisons.

#### Program evaluation

The scores for the evaluation of the program as a whole were 4.2 ± 0.7 for effectiveness, 4.2 ± 1 for compatibility to the treated population and 4.5 ± 0.7 for general satisfaction (Supplementary File 2 in Table S2). Similar scores were received for the webinar, workshop, printed patient brochure and dietitian guide separately. Lower scores were received for the digital patient brochure and recipe links. About one third (33.9%, *n* = 37) of the participants reported distributing brochures to at least half of their clients. The use of digital resources was limited (Supplementary File 2).

## Discussion

This is the first study to evaluate an intervention among dietitians aimed to improve knowledge, attitudes and practices pertaining to legume counseling. The intervention was effective in improving dietitians’ self-reported legume counseling practices, attitudes toward legume counseling, knowledge and personal legume consumption.

The current Israeli dietary guidelines recommend daily legume consumption. At baseline, only 29% of the overall sample recommended to most or all their patients (76–100%) to consume legumes daily. The more general recommendation of increasing legume intake is presumably easier to implement; however, even this more acceptable recommendation was provided by only 47% of the dietitians at baseline. These low rates were similar to the those found in a previous Israeli survey (30.6 and 47.7%, respectively) (35) and further affirm the necessity of the current intervention.

The intervention was effective in improving legume recommendations for daily consumption or more generally to increase consumption. For the control group, the pairwise comparison identified divergent responses. A possible explanation for the observed decline in counseling was that the follow-up survey was administered at the end of the summer, while the baseline survey was completed in the spring. Legumes are more typically consumed in the winter in dishes like soups and casseroles (45–47); therefore, dietitians may tend to decrease their legume counseling to patients during the summer. The decrease in counseling in the intervention group was negligible and significantly lower than in controls, indicating that the intervention was effective in mitigating a reduction in counseling that might be attributed to seasonality. In the repeated measures model, both groups showed a significant improvement, albeit a higher improvement for the intervention group. A possible explanation for the improvement reported by the control group is that more than half of the dietitians were not aware of the current recommendation of daily legume intake. The invitation to participate in the study highlighted this recommendation, which may have impacted the control group directly. Lastly, although the intervention group was specifically asked not to share the contents of the intervention with other dietitians, the possibility of contamination cannot be ruled out; particularly since Israel is a small country, where it is common for dietitians to work simultaneously in several places.

Findings also show that the intervention was effective in improving knowledge and attitudes in regard to legume counseling. Specifically, the largest improvement was seen for “having adequate resources.” All of the resources received high scores regarding general satisfaction. The printed brochure and dietitian guide received high scores also for effectiveness and compatibility for the treated population. The digital materials scored lower in these two parameters, which was reflected in the lower rate of dietitians reporting using them. Future cooperation with HMOs and hospitals is recommended, including integration into their computerized systems, which might potentially encourage more widespread use of the digital materials, thus supporting effective, low-cost legume counseling for the long-term.

Although the legume perception scores improved significantly in the intervention group, and no improvements were observed in controls, the interaction between time and the group was not significant, i.e., the intervention group perceptions did not improve significantly in comparison to the control. It is possible that the sample size was not adequate to detect relatively smaller changes that occurred in legume perceptions. Similarly, the sample size might have precluded finding statistical significance in regard to changes in participant’s personal legume consumption, despite seeing a trend toward improvement.

The attitude score regarding sustainability was the only score that also improved in the control group. It is possible that the survey in itself served as a form of intervention, alerting participants to this dimension (21). The overall high agreement with the need to integrate the environmental consideration into the dietitian’s practice found in this study is in line with studies conducted among American (48) and European (49, 50) dietitians. Furthermore, professional associations worldwide (32–34, 51–54) have recognized dietitians as key agents for change in the transition toward sustainable food systems. This can be achieved through many venues including counseling for individuals or groups, menu planning and procurement for institutions, advising the food industry and working in public health, policy or academic settings.

### Implication for policy and practice

Dietetic associations have acknowledged the necessity of providing adequate sustainability training and resources for dietitians (32–34, 51–55). This study can potentially serve as a model for similar dietitian training programs in more countries, addressing the gaps identified in existing programs. Dietetic academic education and practical training regarding sustainability were found to be inconsistent and insufficient (30, 56, 57). American programs lack content regarding the linkages between human and planetary health, with only few programs addressing plant-based diets’ key role in dealing with the environmental crisis (57). In addition, creating educational resources regarding legumes may help overcome dietitians’ challenges in promoting sustainable diets that include lack of educational resources (48), particularly regarding sustainable food options and substitutions for use in meal and menu planning (58). It is essential to integrate legumes as a key component in dietetic educational programs as a sustainable, healthful, minimally processed and inexpensive source of protein.

### Strengths and limitations

Several limitations of this study should be noted. Data were self-reported which can lead to social desirability bias. As this study focused on dietitians, it is not possible to determine if the reported changes in counseling practices modified patients’ legume intake. The follow-up lasted 3 months, thus the intervention’s long-term effects are not known. Future studies should include a long-term follow-up. It is likely that participants were initially more interested in the topic of legumes; additionally, recruitment through professional associations and social media may have favored more engaged dietitians, potentially introducing selection bias and limiting generalizability. Nonetheless, the age and sex distribution of our sample was very similar to that of the Israeli dietitian population indicating representativity in those regards (personal communication, Ministry of Health). In addition, the sample included dietitians from varied workplaces and from all geographical regions in Israel. Lastly, present findings may be more transferable to countries where guidelines recommend legumes as a preferred protein source [e.g., Canada (17), New Zealand (59)], as national dietary guidelines supporting sustainable diets facilitate dietitians’ promotion of such patterns (49). The intervention was designed to be short, online, and low-cost. In contexts without such guidelines, or for other health professionals, a more comprehensive intervention may be required. The strengths of this study include its novelty as the first study to evaluate an intervention aiming to improve dietitians’ legume counseling practices, and being the first randomized controlled trial performed in high-income countries evaluating a legume promotion intervention. An additional strength is the use of validated behavior change models in the intervention design.

## Conclusion

The present study suggests that low-cost, online training can improve dietitians’ legume counseling practices, attitudes, knowledge and personal legume consumption in the short term. Promoting legume consumption as a sustainable and healthy protein source is an urgent task of global importance, in which dietitians can act as key agents for change through their wide circles of influence.

## Data Availability

The raw data supporting the conclusions of this article will be made available by the authors, without undue reservation.
