# Supplementary material for: Dietitians as agents of change to increase legume consumption: a randomized controlled trial of a behavioral intervention
Source: Front Nutr. 2026 Jan 28;12:1713719. doi: 10.3389/fnut.2025.1713719 (PMC12890693; doi:10.3389/fnut.2025.1713719)
Supplement: Supplementary file 1 [file Table_1.docx]

**Supplementary File 1a**

**Content of the Patient Brochure**

a) Photos of common legume varieties available in Israel. b) Explanations of the nutritional, health, food security and environmental benefits of legumes. c) Answers to misconceptions regarding soy. d) Ways of incorporating legumes into breakfast, lunch and dinner (the brochure’s digital version provided links to over 30 recipes). e) Instructions on how to cook legumes. f) Tips for quick methods of preparation. g) Approaches to improve digestion. h) Link and QR code to the brochure’s digital version: <https://efsharibari.health.gov.il/media/3103/legumes-flier.pdf>

**Supplementary File 1b**

**Legumes- knowledge, attitudes and counselling habits among dietitians**

This survey takes approximately 10-15 minutes to complete. You are free to choose not to answer all the questions in the survey and to stop at any time.

The survey includes four sections: personal details, questions regarding legume counselling practice and attitides, knowledge questions and personal legume intake.

We thank you for your cooperation,

**Personal details**

**Gender**

- female

- male

- other

- prefer not to answer

**Year of birth**

**Education**

Bachelor’s degree

Master’s degree

Doctoral degree

**Where is your primary workplace (the place you work the most hours in a typical week) ?**

Hospital

Health Maintenance Organization (HMO)

Retirement Home

Private Clinic

Gym

Private Institute

Food Industry

Public Health

University / Research Institute

Other

**Which conditions (or population groups) do you most often treat? (please mark up to 4 items)**

obesity

diabetes

cardiovascular diseases

gastroenterology

nephrology

oncology

eating disorders

healthy eating promotion

vegetarian and vegan diets

infants and children

pregnancy and lactation

athletes

bariatrics

surgery

geriatrics

other

**How many years have you worked as a dieititan?**

**How many patients do you see in an average week?**

**What is the average number of sessions for each patient?**

**Do you also provide virtual counselling meeting (e.g., via Zoom, WhatsApp, etc.)?**

**Questions regarding legume counselling practice and attitides**

**I recommend consumption of legumes on a daily basis:**

1. To almost all of my patients (76%-100% of my patients)

2. To most of my patients (51%-75% of my patients)

3. To some of my patients (26%-50% of my patients)

4. To a few of my patients (up to 25% of my patients)

5. To none of my patients (0% of patients)

**I recommend consumption of legumes on a daily basis to patients who are vegan\vegetarian or rarely eat meat:**

1. To almost all of my patients (76%-100% of my patients)

2. To most of my patients (51%-75% of my patients)

3. To some of my patients (26%-50% of my patients)

4. To a few of my patients (up to 25% of my patients)

5. To none of my patients (0% of patients)

**I recommend that patients increase their legume consumption:**

1. To almost all of my patients (76%-100% of my patients)

2. To most of my patients (51%-75% of my patients)

3. To some of my patients (26%-50% of my patients)

4. To a few of my patients (up to 25% of my patients)

5. To none of my patients (0% of patients)

Please indicate your level of agreement with the following statements:

*Scale of 1-5: strongly disagree (1 point)- strongly agree (5 points)*

Note: The following ten statements were later reduced by factor analysis to three factors: 'Resources, confidence and knowledge'; 'Importance, effectiveness and time'; 'Sustainability'.

**Counselling patients regarding legume consumption is important for me.** (Importance, effectiveness and time)

**Counselling patients** **regarding legume consumption is important for my colleagues.** (Importance, effectiveness and time)

**I have sufficient knowledge to counsel patients regarding legume consumption.** (Resources, confidence and knowledge)

**I am not certain in what context it would be suitable to bring up the topic of** **legume consumption** **in a counselling session.** (Importance, effectiveness and time)

**I am confident in my ability to** **counsel patients regarding legume consumption.** (Resources, confidence and knowledge)

**I have** **enough time to counsel patients regarding legume consumption.** (Importance, effectiveness and time)

**I have adequate didactic resources to counsel patients regarding legume consumption.** (Resources, confidence and knowledge)

**In my opinion, counselling patients regarding legume consumption is ineffective (does not result in higher consumption).** (Importance, effectiveness and time)

**Dietary choices have a significant impact on the environment (i.e., greenhouse gas, water and soil resources).** (Sustainability)

**T****he environmental factor should become one of the** **dietitian's considerations when consulting patients.** (Sustainability)

. Please indicate your level of agreement with the following statements:

*Scale of 1-5: strongly disagree (1 point)- strongly agree (5 points)*

Note: The following ten statements were later reduced by factor analysis to three factors: 'Adequate protein and iron source'; 'Management of consumption barriers'; 'Weight and glucose management'.

**I perceive legumes to be:**

**Satiating** (Weight and glucose management)

**Suitable for persons with diabetes** (Weight and glucose management)

**A good source of iron** (Adequate protein and iron source)

**A good source of protein** (Adequate protein and iron source)

**Hard to digest** (Management of consumption barriers)

**Not suitable for children** (Adequate protein and iron source)

**Promotors of weight loss** (Weight and glucose management)

**A replacement for a meat dish** (Adequate protein and iron source)

**A cause of intestinal gas** (Management of consumption barriers)

**Time consuming to prepare** (Management of consumption barriers)

**Knowledge questions**

**The amount of protein in one egg is similar to that of half a cup of cooked chickpeas** True\ False\ Don't know

**The amount of iron in half a cup of cooked beans is higher than that of 150 gr of cooked turkey breast** True\ False\ Don't know

**Legumes are an environmentally friendly source of protein in comparison to animal protein** True\ False\ Don't know

**Legumes may help reduce LDL cholesterol levels** True\ False\ Don't know

**Dietary guidelines worldwide define legumes as an inferior protein source in comparison to animal protein** True\ False\ Don't know

**It is necessary to sprout legumes in order to receive their nutritional benefits** True\ False\ Don't know

**It is not necessary to combine legumes and grains in a single meal in order to obtain high quality protein** True\ False\ Don't know

**Questions regarding personal consumption patterns**

**How often do you consume legumes (not including soy milk in coffee)*?***

- Once a month or less

- 2–3 times per month

- Once a week

- 2-3 times per week

- 4-6 times per week

- Every day

**Do you define yourself as a vegetarian or a vegan?**

- No

- No, but I try to reduce my meat intake

- Yes, vegetarian

- Yes, vegan

**The current Israeli Dietary Guidelines' recommendation of daily legume intake was emphasized in the publicity materials of the intervention program. Were you aware of this recommendation before being exposed to these materials?**

**The intervention group follow up survey included the following additional section evaluating the training program:**

**Did you watch the webinar?**

- Yes, I watched it fully until the end

- Yes, I watched most of it (30 min <)

- Yes, I watched a small part of it (30 min >)

- No, I did not get to watch it

**After the program had been completed, a recording of the lecture given in the workshop was sent to you for your convenience, did you watch it?**

- Yes, I watched it fully until the end

- Yes, I watched most of it (30 min <)

- Yes, I watched a small part of it (30 min >)

- No, I did not get to watch it

**Have you handed out the printed brochure to patients?**

1. To almost all of my patients (76%-100% of my patients)

2. To most of my patients (51%-75% of my patients)

3. To some of my patients (26%-50% of my patients)

4. To a few of my patients (up to 25% of my patients)

5. To none of my patients (0% of patients)*

* Those participants were asked to provide the reason for not using the resource (as an open question).

**Have you sent the digital brochure to patients (or directed patients to download it from the website)?**

1. To almost all of my patients (76%-100% of my patients)

2. To most of my patients (51%-75% of my patients)

3. To some of my patients (26%-50% of my patients)

4. To a few of my patients (up to 25% of my patients)

5. To none of my patients (0% of patients)*

* Those participants were asked to provide the reason for not using the resource (as an open question).

**Have you recommended patients to enter the recipe links in the digital brochure?**

1. To almost all of my patients (76%-100% of my patients)

2. To most of my patients (51%-75% of my patients)

3. To some of my patients (26%-50% of my patients)

4. To a few of my patients (up to 25% of my patients)

5. To none of my patients (0% of patients)*

* Those participants were asked to provide the reason for not using the resource (as an open question).

**Have you used the professional guide for dietitians during consultations to aid in visual demonstration of legume dishes and/or legume variety?**

1. To almost all of my patients (76%-100% of my patients)

2. To most of my patients (51%-75% of my patients)

3. To some of my patients (26%-50% of my patients)

4. To a few of my patients (up to 25% of my patients)

5. To none of my patients (0% of patients)*

* Those participants were asked to provide the reason for not using the resource (as an open question).

**Has your participation in the program led to an increase in the amount of time you devote to the subject of legumes during consultation meetings?**

- Yes, in a significant manner

- Yes, slightly

- No

**In your opinion, to what extent has the program and each of its components contributed to improving the effectiveness of your counselling regarding legumes to your patients (i.e., has led to an actual increase in legume intake among your patients)?**

*Scale of 1-5: to a small extent (1 point)- to a great extent (5 points)* Program Webinar Workshop Dietitian guide Patient brochure Digital brochure Recipe links

**To what extent was the program and each of its components compatible with the population you treat?**

*Scale of 1-5: to a small extent (1 point)- to a great extent (5 points)* Program Webinar Workshop Dietitian guide Patient brochure Digital brochure Recipe links

**What is your general satisfaction with the program and each of its components?**

*Scale of 1-5: very low (1 point)- very high (5 points)* Program Webinar Workshop Dietitian guide Patient brochure Digital brochure Recipe links

**We would appreciate if you could share your general feedback regarding the program and suggestions for improvement**
