# Supplementary material for: Dietitians as agents of change to increase legume consumption: a randomized controlled trial of a behavioral intervention
Source: Front Nutr. 2026 Jan 28;12:1713719. doi: 10.3389/fnut.2025.1713719 (PMC12890693; doi:10.3389/fnut.2025.1713719)
Supplement: Supplementary file 2 [file Table_2.docx]

**Supplementary file 2**

**Table S1. Pairwise Comparisons of Dietitians' Legume Recommendations to Patients, (n=212)**

| **p-value*** | **OR (95% CI)** | **Control group n=104 (n,%)** | **Intervention group n=108 (n,%)** |  |
| --- | --- | --- | --- | --- |
| ***Recommending patients to consume legumes daily*** | | | | |
| 0.009 | 3.25 (1.57-7.18) | 11 (10.6%) | 30 (27.8%) | **Remaining in 76%-100% of patients** |
| 0.216 | 1.54 (0.78-3.10) | 17 (16.3%) | 25 (23.1%) | **Improving to 76%-100% of patients** |
| 0.041 | 0.29 (0.09-0.77) | 15 (14.4%) | 5 (4.6%) | **Declining to 75% or less of patients** |
| 0.052 | 0.56 (0.33-0.97) | 61 (58.7%) | 48 (44.4%) | **Remaining in 75% or less of patients** |
| ***Recommending patients to increase legume intake*** | | | | |
| 0.049 | 1.94 (1.09-3.48) | 28 (26.9%) | 45 (41.7%) | **Remaining in 76%-100% of patients** |
| 0.390 | 1.33 (0.70- 2.59) | 20 (19.2%) | 26 (24.1%) | **Improving to 76%-100% of patients** |
| 0.049 | 0.36 (0.14-0.83) | 19 (18.3%) | 8 (7.4%) | **Declining to 75% or less of patients** |
| 0.228 | 0.66 (0.37-1.19) | 37 (35.6%) | 29 (26.9%) | **Remaining in 75% or less of patients** |

*The p-values presented in the table are adjusted for multiple comparisons.

**Process Evaluation by the Intervention Group (n=109)**

1. **Use of the program components**

For the first question “Did you watch the webinar", almost all (95.4%, n=104) reported watching the webinar until the end. Less than 3% (n=3) reported watching most of it (more than 30 minutes), and 1.8% (n=2) reported watching only a small part of the webinar (less than 30 minutes). None of the participants reported not watching the webinar at all.

For the second question regarding watching the workshop recording, more than half (53.3%, n=57) reported not watching the recording at all. Nearly a third (32.7%, n=35) watched the recording until the end, with 9.3% (n=10) and 4.7% (n=5) reporting watching most of it, or a small part of it, respectively.

Regarding handing out the printed brochure to patients, only 14 (12.8%) reported handing it out to more than 76% of their patients. Almost the same number (n=15, 13.8%) reported not providing the brochure to any of their patients, with the main reasons reported that the brochure was not relevant to their work because they provide virtual consultation (n=4) or because of their patient population (e.g., children, English speakers) (n=4); an additional n=4 reported forgetting to bring the brochures to the clinic or not being used to handing out material.

Regarding sending the digital brochure to patients (or directing them to download it from the website), only 8 (7.3%) reported sending it to more than 76% of their patients, and 48 (44%) reported not sending it to any of their patients, with the main reasons reported that they preferred to hand out the printed brochure (n=17); they forgot that a digital version of the brochures was also provided to them (n=9); they work in Health Maintenance Organizations (HMOs), and do not have the option of emailing exterior materials to patients (n=7); an additional n=7 reported that it was not relevant for their treated population (e.g., older persons, ultra-orthodox Jews).

Regarding recommending patients to enter the recipe links in the digital brochure, only 14 (12.8%) reported recommending it to more than 76% of their patients. Approximately a third (n=37, 33.9%) reported not recommending it to any of their patients, with the main reasons reported that they forgot (n=14); they have their own recipes that they are used to give to patients (n=10), and n=7 reported that it was not relevant for their treated population (e.g., dialysis patients, older persons, ultra-orthodox Jews).

Regarding using the professional guide for dietitians during consultations (to aid in visual demonstration of legume dishes and/or legume variety) only 10 (9.3%) reported using it with more than 76% of their patients. Approximately a quarter (n=26, 24.1%) reported not using it with any of their patients, with the main reasons reported that they did not see a need for it (n=11); they used the patient brochure (n=5) and that they forgot (n=4).

**2. Evaluation of the program components** Table S3 provides the rating of the participants for the intervention as a whole, and each of the intervention components, on a Likert scale of 1-5, in regard to their impact on improving legume counselling effectivity, compatibility with their patient population, and general satisfaction.

| **Table S2. Participants' Evaluation of the Program Components (n=109)** | | | |
| --- | --- | --- | --- |
| **General satisfaction^3^  (mean ± SD)** | **Compatibility to the treated population^2^ (mean ± SD)** | **Improving legume counselling effectivity^1^**  **(mean ± SD)** |  |
| 4.5±0.7 | 4.2±1 | 4.2±0.7 | **Program as a whole** |
| 4.5±0.7 | 4.1±1 | 4.1±0.9 | **Webinar** |
| 4.5±0.7 | 4.2±1 | 4.2±0.8 | **Workshop** |
| 4.5±0.8 | 4.1±1.2 | 4.0±1.1 | **Dietitian guide** |
| 4.5±0.7 | 4.2±1.1 | 4.1±1.0 | **Patient brochure** |
| 4.2±1.0 | 3.7±1.4 | 3.5±1.3 | **Digital brochure** |
| 4.3±0.9 | 3.8±1.4 | 3.6±1.2 | **Recipe links** |
| 5-point Likert scale from " to a small extent " (1) to "to a great extent" (5)  ^1^"In your opinion, to what extent has the program and each of its components contributed to improving the effectiveness of your counselling regarding legumes to your patients (i.e., has led to an actual increase in  legume intake among your patients)?" ^2^ "To what extent was the program and each of its components compatible with the population you treat?" ^3^ "What is your general satisfaction with the program and each of its components?" | | | |

Almost all reported that the participating in the program increased the amount of time they devote to legumes during consultations, with 37.6% (n=41) reporting the time devoted to legume counselling increased in a significant manner, and 56.9% (n=62) reported it increased slightly. Only n=6 (5.5%) reported that the time they devoted to this topic has not increased.

Half of the participants (N=59, 54%) provided also general feedback and suggestions for improvement. Most provided overall high positive feedback, e.g., "The program was exceptional ", "It was very interesting and enjoyable", "The program should become a part of BSc. Nutritional Science curriculum", "I don't know whether there is something to improve in the program, in my opinion it was excellent ". Few provided specific examples of how the program was successful. For example, a few mentioned that the program has led to a higher personal or household legume consumption, or that there was high compliance from patients in regard to incorporating legumes in their diets, and very positive feedback from patients regarding the brochure.

A few made specific suggestions for improvement of the intervention: 1) incorporating the digital brochure within the HMOs software to enable sending it directly to patients; 2) adding additional reminders; 3) additional content regarding specific considerations for children and for diabetic patients; and 4) adding a cooking workshop/ having one of the sessions as a face-to-face meeting.
